# Autophagy Dysfunction in ALS: from Transport to Protein Degradation

**DOI:** 10.1007/s12031-022-02029-3

**Published:** 2022-06-16

**Authors:** Marta Cozzi, Veronica Ferrari

**Affiliations:** Dipartimento Di Scienze Farmacologiche E Biomolecolari, Università Degli Studi Di Milano, 20133 Milan, Italy

**Keywords:** Amyotrophic lateral sclerosis, Autophagy, Mutations, Animal models

## Abstract

Amyotrophic lateral sclerosis (ALS) is a fatal neurodegenerative disease affecting upper and lower motor neurons (MNs). Since the identification of the first ALS mutation in 1993, more than 40 genes have been associated with the disorder. The most frequent genetic causes of ALS are represented by mutated genes whose products challenge proteostasis, becoming unable to properly fold and consequently aggregating into inclusions that impose proteotoxic stress on affected cells. In this context, increasing evidence supports the central role played by autophagy dysfunctions in the pathogenesis of ALS. Indeed, in early stages of disease, high levels of proteins involved in autophagy are present in ALS MNs; but at the same time, with neurodegeneration progression, autophagy-mediated degradation decreases, often as a result of the accumulation of toxic protein aggregates in affected cells. Autophagy is a complex multistep pathway that has a central role in maintaining cellular homeostasis. Several proteins are involved in its tight regulation, and importantly a relevant fraction of ALS-related genes encodes products that directly take part in autophagy, further underlining the relevance of this key protein degradation system in disease onset and progression. In this review, we report the most relevant findings concerning ALS genes whose products are involved in the several steps of the autophagic pathway, from phagophore formation to autophagosome maturation and transport and finally to substrate degradation.

## Introduction

### Amyotrophic Lateral Sclerosis

Amyotrophic lateral sclerosis (ALS) is a rare adult motor neuron disease (MND) characterized by degeneration of upper and lower motor neurons (MNs), leading to progressive muscle atrophy and death within 3–5 years after symptom onset. Besides MNs, skeletal muscle (Dobrowolny et al. 2008; Onesto et al. 2011; Cicardi et al. 2018; Meroni et al. 2019) and glial cells (Trotti et al. 1999; Lobsiger et al. 2009; Philips et al. 2013) can be targeted by ALS. Neurons residing in the frontotemporal cortex might be affected, too, which may lead to the development of a mixed form of MND and frontotemporal dementia (FTD) referred to as ALS/FTD (Robberecht and Philips 2013). No drugs are currently available to treat or cure ALS (Taylor et al. 2016).

Most ALS cases (90%) are sporadic (sALS), while only 10% are characterized by familial inheritance (fALS). Clinical symptoms fully overlap between the two forms of the disease, but fALS tends to be more severe compared to sALS. Inherited ALS cases are associated to both loss-of-function (LOF) and aberrant gain-of-function (GOF) mutations, as well as to mixed LOF and GOF, that can be found in more than 40 genes. Nonetheless, around 30% of fALS genetic causes still need to be identified, indicating that ALS is a disease with a widely heterogeneous genetic background (Cristofani et al. 2020). Noteworthily, the wild-type forms of the gene products causing fALS display an aberrant behavior in sALS too, suggesting that the two forms of the disease probably share some pathogenetic mechanisms (Neumann et al. 2006).

ALS is caused by a combination of genetic, epigenetic, and environmental factors which result in different pathogenetic mechanisms capable of triggering neuronal damage (Morgan and Orrell 2016). To date, the precise etiology of ALS is still unknown, as well as the exact molecular mechanisms involved in the degeneration of motor neurons (Shaw 2005). The analysis of ALS-related genes outlined the main pathophysiological mechanisms involved in neurodegeneration in sALS and fALS: oxidative stress, mitochondrial dysfunction, impairment of axonal transport, excitotoxicity, protein aggregation, endoplasmic reticulum (ER) stress, abnormal RNA processing, and neuroinflammation (Kiernan et al. 2011; Taylor et al. 2016). One of the main disease mechanisms is the alteration in protein quality control (PQC). In this respect, a distinctive hallmark of both fALS and sALS is the formation of aberrant aggregates of TAR DNA-binding protein 43 (TDP-43), often together with a mixture of other proteins, into target cells. The only exceptions are fALS forms linked superoxide dismutase 1 (SOD1) mutation, being the only devoid of TDP-43 inclusions. When mutated, most of the proteins characterizing the aggregates misfold and partition into initially small structures which later degenerate into aggresomes and insoluble inclusions if they are not rapidly degraded (Patel et al. 2015; Boeynaems et al. 2017). The accumulating aggregates end up tampering with the PQC system, that fails in keeping under control protein misfolding and undergoes saturation, leading inclusions to become toxic for the affected cells.

In this review, we will focus on the connections between ALS and autophagy, one of the main branches of the PQC system. To highlight the strict relations between ALS and autophagy and to better clarify the role of ALS-associated autophagic genes, we will describe the function and report the most relevant findings on the pathogenetic mechanisms lying behind the mutations of these genes in ALS (Table 1).

**Table 1:** Summary of autophagic genes role in ALS pathogenesis. *LOF =* loss of function, *GOF* = gain of function

Gene	Role in autophagy and in ALS pathogenesis	References
*ALS2*	Regulates endosomal maturation → truncating mutations cause rapid ALS2 degradation and abolish its guanine-nucleotide exchange factor activity for RAB5, leading to impairments in endosome maturation (LOF)	Hadano et al. (2001); Yamanaka et al. (2003); Cai et al. (2005)
*C9ORF72*	Regulates autophagy initiation and maturation → G4C2 hexanucleotide expansion leads to dipeptide repeats accumulation (GOF) and impaired ULK1 complex formation (LOF)	DeJesus-Hernandez et al. (2011); Koppers et al. (2015)
*CHMP2B*	Modulates ESCRT-III complex assembly to form multivesicular bodies → truncating mutations disrupt autophagosome-lysosome fusion (GOF)	Lee et al. (2007); Han et al. (2012); West et al. (2020)
*DCTN1*	Essential cofactor in dynein-mediated retrograde transport of autophagosomes and lysosomes → mutations are associated to decreased DCTN1 levels and immature autophagosome accumulation (LOF); protein accumulation (GOF) is still debated	Lai et al. (2007); Laird et al. (2008); Ikenaka et al. (2013); Stockmann et al. (2013)
*FIG4*	Regulates PI(3,5)P2 levels together with PIKfyve and VAC14 to modulate late endosome maturation → mutations lead to enlarged endosomes accumulation (LOF)	Chow et al. (2007); Ferguson et al. (2009); Bharadwaj et al. (2016)
*KIF5A*	Transports lysosomes along axons → mutations might impair the autophagic flux (LOF)	Liu et al. (2021); Baron et al. (2022)
*OPTN*	Autophagy receptor → mutations prevent association with ubiquitinated substrates, including mitochondria, and disrupt myosin VI-mediated autophagosome-lysosome fusion (LOF)	Maruyama et al. (2010); Korac et al. (2013); Sundaramoorthy et al. (2015)
*SQSTM1/p62*	Autophagy receptor → mutations prevent association with ubiquitinated substrates or interaction with LC3-II (LOF)	Le Ber et al. (2013); Teyssou et al. (2013); Lattante et al. (2015); Goode et al. (2016)
*TBK1*	Activates autophagy receptors through phosphorylation and promotes autophagosome formation and maturation → truncating mutations reduce TBK1 levels and abolish autophagy receptors activation (LOF)	Ryzhakov and Randow (2007); Freischmidt et al. (2015); Brenner et al. (2019); Duan et al. (2019)
*TUBA4A*	Forms microtubules → mutations alter microtubule stability, therefore tampering with microtubule-based transport of autophagosomes and lysosomes (LOF), and lead to TUBA4A aggregation (GOF)	Howes et al. (2014); Rademakers and van Blitterswijk (2014); Smith et al. (2014)
*UBQLN2*	Promotes the autophagic disposal of ubiquitinated ER proteins and regulates autophagy initiation and lysosomal acidification → mutations disrupt substrate recognition and increase autophagy activation while impairing lysosome-mediated degradation (LOF); UBQLN2 aggregation (GOF) is still debated	Deng et al. (2011); Wu et al. (2015); Şentürk et al. (2019)
*VAPB*	Promotes autophagy and mitophagy initiation → mutations alter the autophagic flux (LOF); VAPB aggregation (GOF) is still debated	Teuling et al. (2007); Kuijpers et al. (2013a)
*VCP*	Promotes the autophagic disposal of ubiquitinated substrates and aggresomes, operates in autophagy initiation and maturation, and regulates lysosome homeostasis → mutations aberrantly trigger autophagy but disrupt autophagosome-lysosome fusion (LOF)	Watts et al. (2004); Johnson et al. (2010); Nalbandian et al. (2012)

### Autophagic Pathway

Autophagy is an evolutionarily conserved cellular mechanism that disassembles old, unnecessary, or dysfunctional cytosolic components and allows their degradation by lysosomal hydrolases to fuel bioenergetic metabolism and repair processes (Klionsky et al. 2021a). Autophagy contributes to the maintenance of proteostasis by avoiding the accumulation of potentially dangerous protein species (Li et al. 2012; Cuervo and Wong 2014; Sica et al. 2015). An active and functional autophagy in neurons is more relevant than in other cell types. Indeed, neurons are cells with no mitotic activity and a very low capacity to regenerate. Moreover, neurons present a particular morphology composed by long axons that makes necessary a characteristic organization for autophagic degradation of synaptic substrates (Cai and Ganesan 2022). Disruption of the autophagic flux occurs in several ALS forms and leads to the accumulation of toxic, ubiquitin-positive inclusion bodies and aberrant stress granules that eventually cause neuronal death (Buchan et al. 2013; Iguchi et al. 2016; Chitiprolu et al. 2018).

Autophagy includes three distinct pathways that promote and regulate the degradation of substrates via lysosomes, microautophagy (Schuck 2020), chaperone-mediated autophagy (CMA) (Kaushik and Cuervo 2018), and macroautophagy, that includes the chaperone assisted–selective autophagy or CASA (Cristofani et al. 2020). Here we will focus on macroautophagy (hereafter autophagy).

**Fig. 1:**
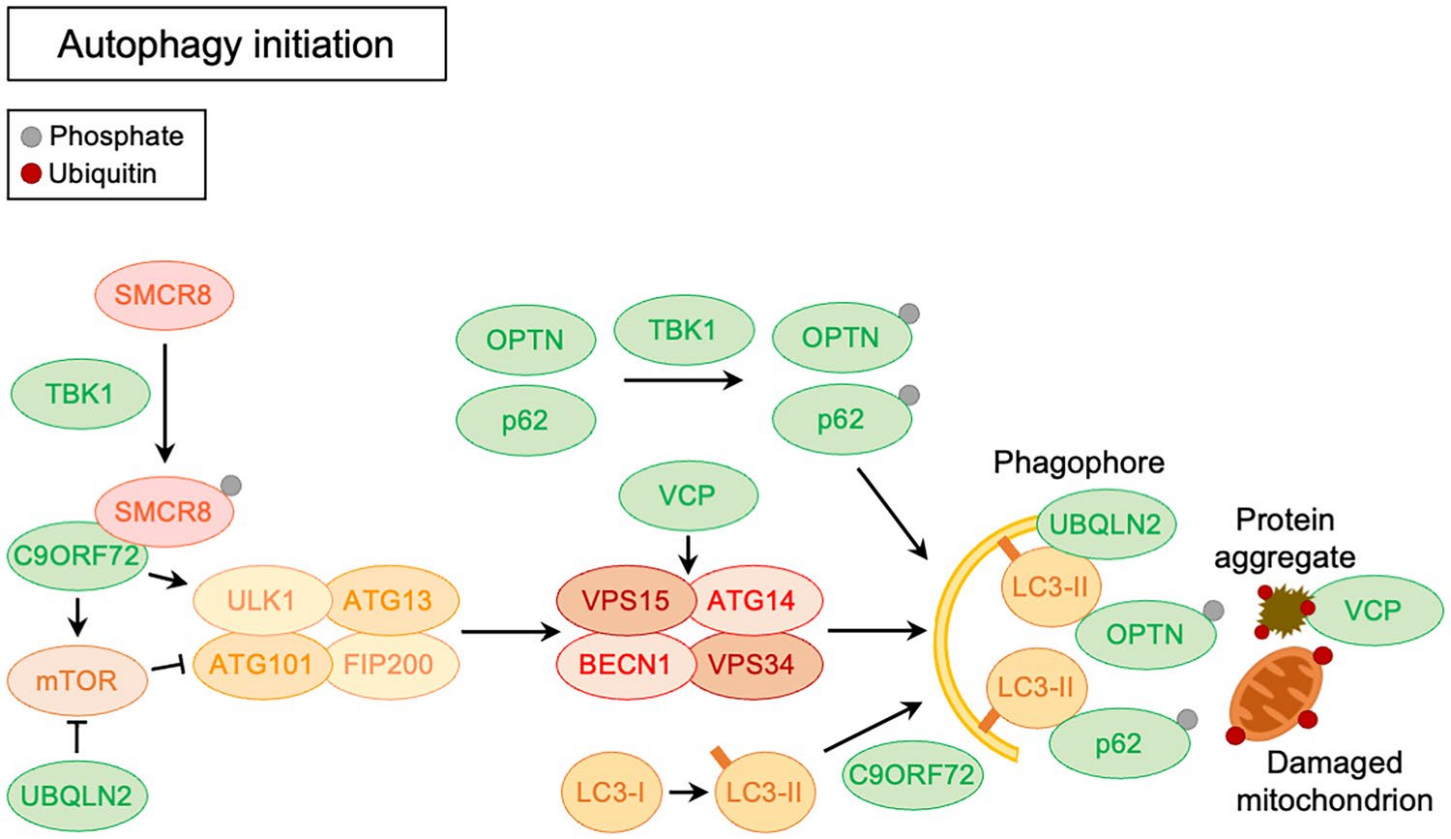
Schematic representation of the ALS-related gene products involved in autophagy initiation (green) in their conventional intracellular functions

Autophagy is characterized by different steps: initiation, elongation, maturation, and degradation (Klionsky et al. 2021b). The initiation consists in the nucleation of the autophagic membrane (phagophore) and is regulated by different complexes. The first complex involved is the preinitiation complex, that is negatively regulated by the mammalian target of rapamycin (mTOR) pathway and is positively regulated by the AMP-activated protein kinase (AMPK) pathway. It is composed by autophagy-related proteins 13 and 101 (ATG13, ATG101), unc-51 like kinase 1/2 (ULK1/2), and FAK family-interacting protein of 200 kDa (FIP200). Another complex is the phosphoinositide 3-kinase (PI3K) complex composed by ATG14, vacuolar protein sorting-associated proteins 34 and 15 (VPS34, VPS15), and Beclin1 (BECN1) (Matsunaga et al. 2010; Cicchini et al. 2015). The contribution of ALS-related genes in this step is represented in Fig. 1.

**Fig. 2:**
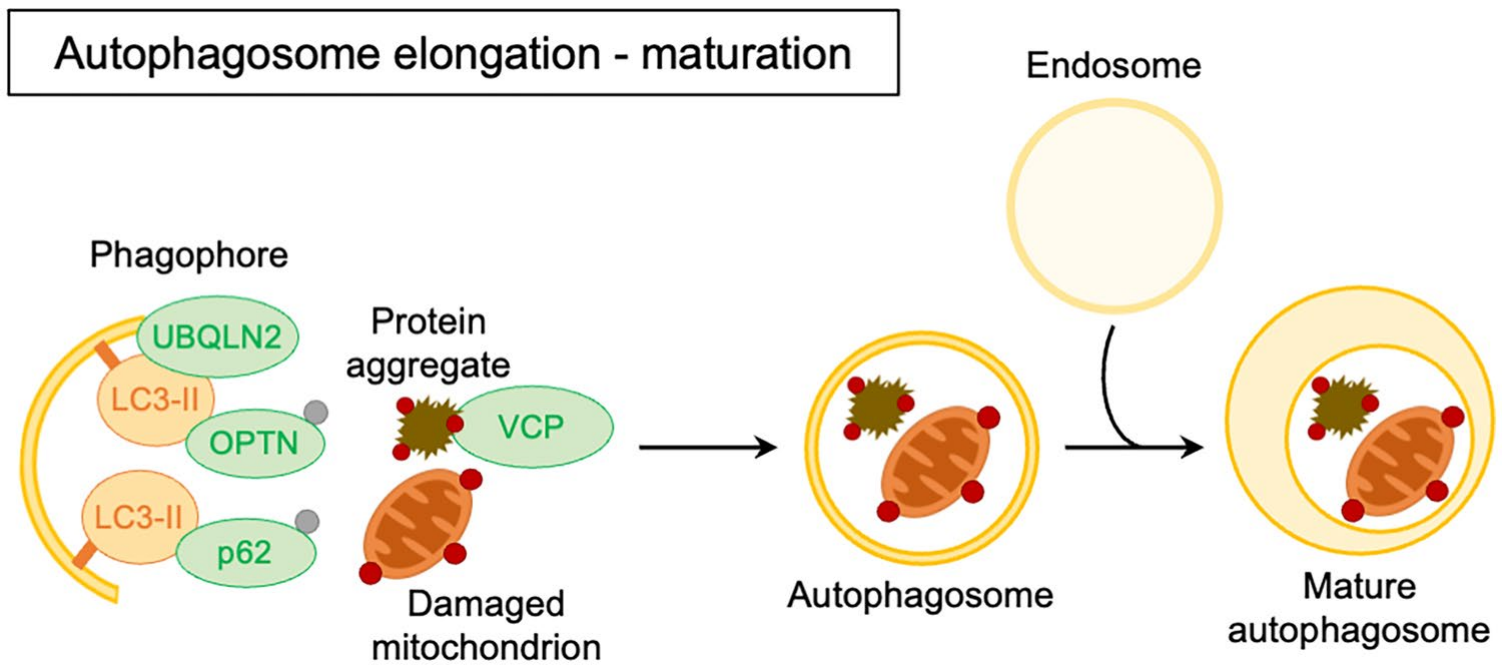
Schematic representation of the ALS-related gene products involved in autophagosome elongation and maturation (green) in their conventional intracellular functions

**Fig. 3:**
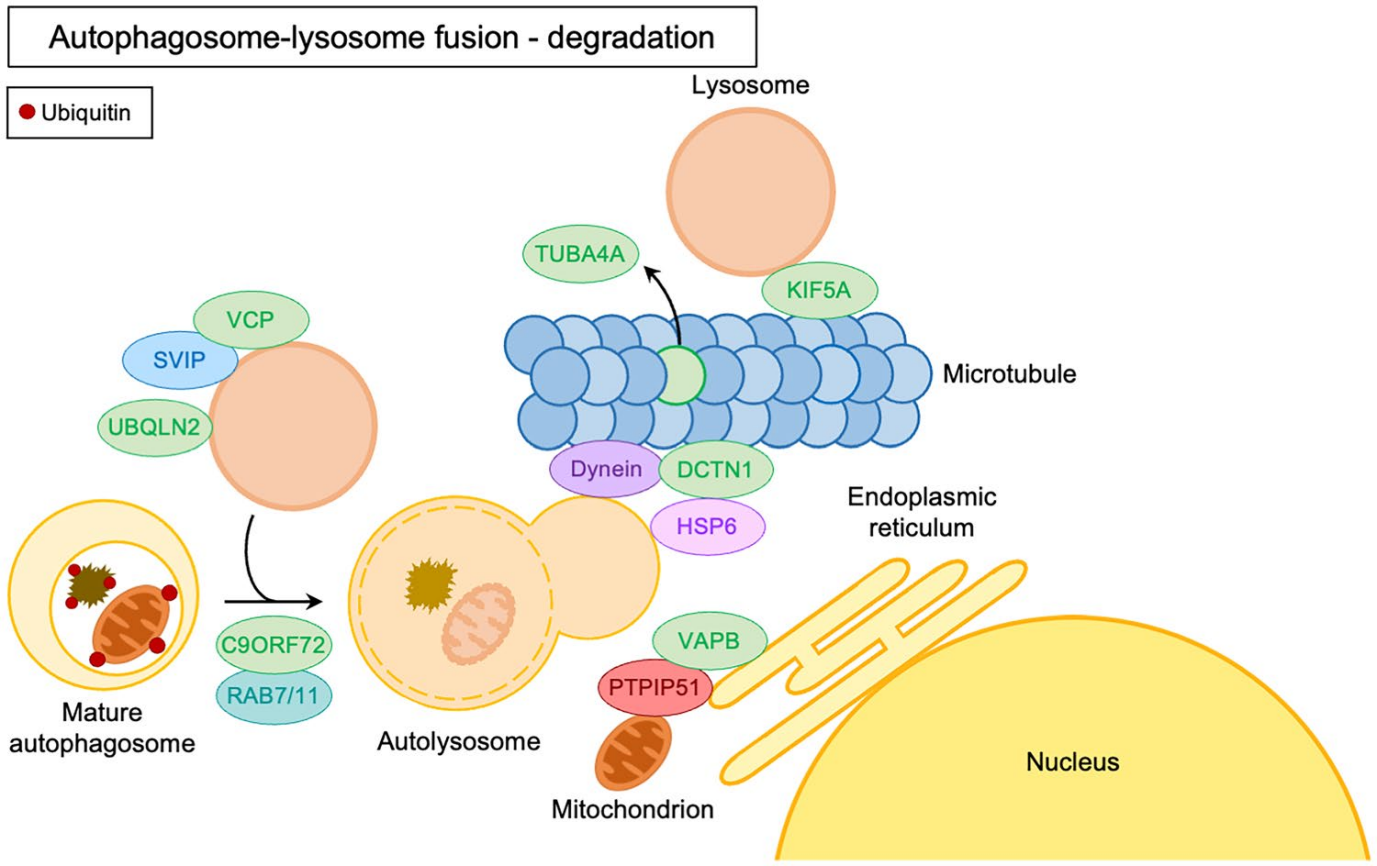
Schematic representation of the ALS-related gene products involved in autophagosome-lysosome fusion and degradation (green) in their conventional intracellular functions

On the forming phagophore are recruited other complexes that regulate its elongation and expansion into the autophagosome. These complexes are the ATG7-ATG3-microtubule-associated protein 1A/1B light chain 3B (MAP1LC3B, or simply LC3) complex that promotes the formation of the lipidated active form of LC3 (LC3-II), and the ATG12-ATG5-ATG16L1 complex (Itakura and Mizushima 2010). Autophagic substrate recognition, targeting, and engulfment are highly regulated. Ubiquitinated misfolded proteins and aggregates are specifically recognized by a complex of chaperones and co-chaperones known as the CASA complex. The components of this complex are heat shock protein B8 (HSPB8), Bcl2-associated athanogene 3 (BAG3), heat shock protein 70 (HSP70), and C-terminus of HSC70-interacting protein (CHIP). The substrates are recognized by HSPB8 and BAG3, and subsequently ubiquitinated by CHIP. The CASA complex is then routed to the forming autophagosome through a dynein-mediated process regulated by BAG3-dynein interaction (Carra et al. 2008). Ubiquitinated substrates are recognized by autophagy receptors such as sequestosome 1 (SQSTM1/p62) or optineurin (OPTN) (Kraft et al. 2010). These proteins act as shuttles mediating substrates to phagophores. Indeed, autophagy receptors, thanks to the specific motifs present in their sequence such as LC3-interacting regions (LIRs), bind components of the autophagic machinery present in the forming autophagosome, such as LC3-II (Rogov et al. 2014). Subsequently to substrate engulfment, the constituted autophagosome maturation may include fusion with an endosome and always terminates in fusion with a lysosome (Zhong et al. 2009). The importance of various ALS-associated genes in these steps has been highlighted in Figs. 2 and 3.

**Fig. 4:**
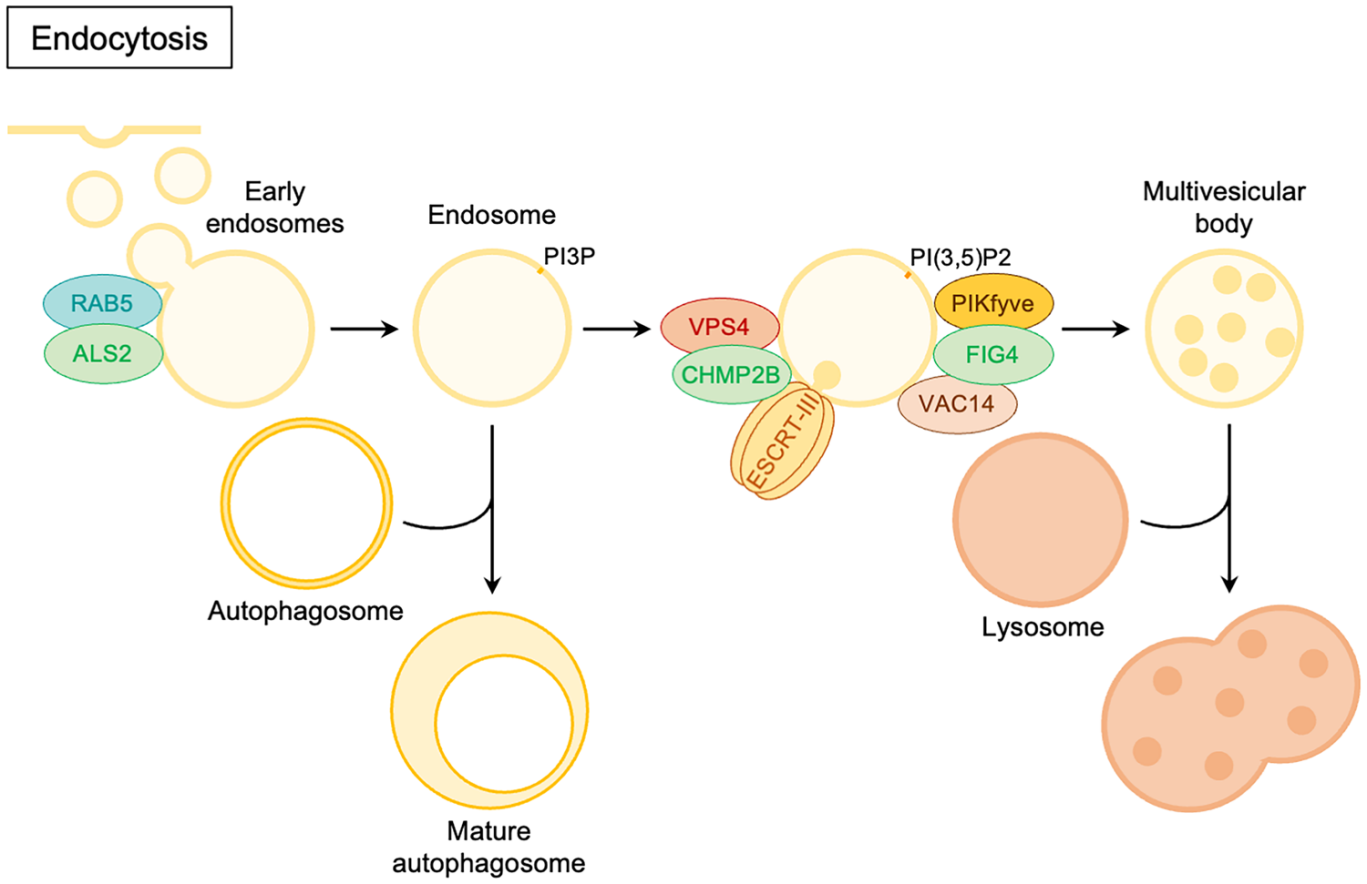
Schematic representation of the ALS-related gene products involved in endocytosis (green) in their conventional intracellular functions

Strictly correlated to autophagy is the endocytosis pathway where the ALS-related genes described in Fig. 4 exert their function. Indeed, the formed endosome as previously described fuses with autophagosomes contributing to its maturation. The ALS-related genes contribution will be better explained in the dedicated sections.

In neurons, axonal transport plays a relevant role in promoting autophagosome maturation by guiding membrane fusion between autophagy intermediates and components of the endolysosomal pathway (Hollenbeck 2014). Fusion with lysosomes eventually results in the degradation of substrates, thanks to hydrolytic enzymes present in the lysosome lumen (Xu and Ren 2015).

## Autophagic Genes Involved in ALS

### ALS2

Alsin (ALS2) is a guanine-nucleotide exchange factor (GEF) encoded by the gene *ALS2* (chromosome 2q33). It is found in all tissues but particularly in neurons (Hadano et al. 2001; Otomo et al. 2003; Yamanaka et al. 2003). ALS2 comprises three GEF-specific domains: an N-terminal regulator of chromatin condensation–like domain (RCC1-like domain, or RLD), a central Db1-pleckstrin homology (DH/PH) domain, and a C-terminal vacuolar protein sorting 9 (VPS9) domain (Hadano et al. 2001; Yang et al. 2001).

ALS2 VPS9 domain mediates its homo-oligomerization and endosomal localization. ALS2 was indeed demonstrated to localize to structures positive for the early endosomal antigen 1 (EEA1) protein, a marker of early endosomes (Otomo et al. 2003; Topp et al. 2004; Kunita et al. 2004). The VPS9 domain is also essential for ALS2 to act as GEF for the small GTPase RAB5, a key player in endosomal membrane fusion (Fig. 4) (Otomo et al. 2003, 2011; Chandran et al. 2007). Transient ALS2 overexpression was shown to promote endosome enlargement and fusion through a RAB5-dependent mechanism in neuronal and non-neuronal cells, confirming ALS2 role in endosomal maturation (Kunita et al. 2004). Furthermore, *ALS2* mutations were demonstrated to be associated with alterations in amphisome formation, and *ALS2* downregulation was shown to cause a decrease in the autophagic clearance of substrates, suggesting that ALS2 is also involved in autophagosome maturation (Hadano et al. 2010; Otomo et al. 2011, 2012). Additionally, ALS2 was demonstrated to protect cultured murine MNs from the neurotoxic activity exerted by the ALS-related SOD1 mutants A4T, G85R, and G93R. It has been shown that ALS2 exerts such neuroprotective activity by directly binding to mutant SOD1 while contemporarily acting as GEF for the small GTPase Rac1 through its DH/PH domain. Indeed, Rac1 activation upon ALS2 binding triggers an antiapoptotic response mediated by the PI3K Akt3 that preserves neurons from death. Both Rac1 downregulation through RNA interference and deletion of ALS2 DH/PH domain fully abolish such neuroprotective function. Interestingly, ALS2 does not display the same activity against any other proteins implicated in neurodegeneration, such as Alzheimer’s disease-related amyloid-β and presenilin 1/2 mutants (Kanekura et al. 2004, 2005).

*ALS2* mutations causing fALS are characterized by a recessive pattern of inheritance and produce premature stop codons in ALS2 sequence that abolish its VPS9 domain (Hadano et al. 2001; Yang et al. 2001; Kress et al. 2005; Sheerin et al. 2014). Truncated ALS2 isoforms are unstable and get rapidly eliminated, so that ALS2 pool is depleted in neurons harboring *ALS2* mutations (Yamanaka et al. 2003). Such loss of ALS2 function is expected to reduce active RAB5 levels, since ALS2-deprived neurons display decreased motility of RAB5-positive endosomes (Lai et al. 2009). Consistently, neurons isolated from *Als2* knock-out mice show defective RAB5-dependent endosomal maturation leading to the accumulation of large, EEA1-positive vacuoles. The impairments in the early phases of endosomal trafficking and fusion caused by ALS2 loss increase MN susceptibility to oxidative stress and promote astrogliosis in the brain and in the spinal cord of *ALS2*-null mice (Cai et al. 2005; Hadano et al. 2006; Devon et al. 2006). Nonetheless, *Als2* knock-out mice do not display an overt ALS phenotype but are only characterized by subtle impairments in motor function and learning (Cai et al. 2005; Hadano et al. 2006; Devon et al. 2006; Yamanaka et al. 2006). Taken together, these data suggest that ALS2 loss is *per se* insufficient to cause neurodegeneration but that it might be responsible for an increased MN vulnerability.

Regarding ALS2 neuroprotective activity, loss of *Als2* was shown to worsen the accumulation of protein aggregates in H46R SOD1 mice MNs. Indeed, *Als2* knock-out correlated with a reduction in the autophagic clearance of mutant SOD1, as a consequence of impaired autophagosome maturation, and disrupted endosomal trafficking (Hadano et al. 2010). Additionally, ALS2 depletion was reported to exacerbate the effect of SQSTM1/p62 loss in H46R SOD1 mice, enforcing the hypothesis that ALS2 role in endosomal dynamics is essential to support autophagy (Hadano et al. 2016).

### C9ORF72

C9ORF72 protein is encoded by chromosome 9 open reading frame 72 (*C9ORF72*), which presents 10 coding exons and two non-coding exons (Smeyers et al. 2021). *C9ORF72* products are 3 transcript variants: a short transcript V1 and two transcripts V2 and V3 that share the coding of exons 2–11 (DeJesus-Hernandez et al. 2011; Smeyers et al. 2021). Interestingly for the development of animal models, the *C9ORF72* human gene is conserved in primates but has a very low similarity in nematode and no orthologue in *Drosophila* (Therrien et al. 2013; Chen et al. 2017). C9ORF72 expression is found in most tissues including all brain regions, spinal cord, and immune system. At cellular level, C9ORF72 is localized in the cytoplasm or in organelles such as Golgi, mitochondria, lysosome, and other components of the endolysosome pathway. The V1 short-isoform was also found localized in the nuclear membrane (Xiao et al. 2015; Atkinson et al. 2015; Aoki et al. 2017; Chitiprolu et al. 2018; Wang et al. 2021). To date, C9ORF72 has been found implicated in different pathways, including nucleocytoplasmic import, stress granule (SG) formation and degradation or recovery, endosomal trafficking, axon growth, and autophagy regulation (Farg et al. 2014; Zhang et al. 2015; Yang et al. 2016; Amick et al. 2016; Sivadasan et al. 2016; Maharjan et al. 2017).

C9ORF72 regulates autophagy at different steps of its pathway as presented in Figs. 1 and 3. Its role in autophagy is supported by two cofactors, Smith–Magenis chromosome region 8 (SMCR8) and WD repeat domain 41 (WDR41), forming the C9ORF72-complex (Tang et al. 2020; Su et al. 2020). Both C9ORF72 and SMC38 present a differentially expressed in normal and neoplastic cells (DENN) domain which functions as a GEF for RAB GTPases (Levine et al. 2013; Iyer et al. 2018). In autophagy initiation, its role is difficult to discriminate as C9ORF72 interacts with different complexes involved in this step. Firstly, C9ORF72 and SMCR8 interact with different members of the ULK1-complex (Yang et al. 2016). During starvation C9ORF72-complex is recruited by solute carrier family 66 member 1 (SLC66A1) to lysosomes, increasing the interaction with ULK1-complex (Amick et al. 2020). Moreover, the C9ORF72 complex promotes the interaction between RAB1 and ULK1-complex, enhancing its recruitment to forming autophagosomes (Webster et al. 2016). Another sign of C9ORF72-positive regulation of autophagy initiation stands in its interaction with RAB5 which promotes the delivery of the PI3K and the ATG7-ATG5-LC3 complexs to autophagosome (Shi et al. 2018; Bingol 2018). The localization of C9ORF72 in forming autophagosomes was suggested to be mediated by SQSTM1/p62 or OPTN, which bind to C9ORF72-complex and RAB39b, another substrate of the complex (Sellier et al. 2016). While these interactions refer to a positive regulation of C9ORF72 complex in autophagy initiation, another mediates a negative regulation of autophagy. Indeed, signs of the interaction of C9ORF72 with mTOR complex 1 (mTORC1) were detected, which suggests a supporting role in mTORC1 function. *C9ORF72* silencing decreases mTORC1 activity, promoting autophagy transcriptional factor regulators activation (Ji et al. 2017; Wang et al. 2020). C9ORF72 is also implicated in autophagosome maturation mainly by cooperating with RAB proteins, such as RAB7 and RAB11, which regulate microtubular transport of autophagosomes, late endosomes, multivesicular bodies, and the fusion with lysosomes (Hyttinen et al. 2013; Ao et al. 2014; Aoki et al. 2017; Tang et al. 2020).

*C9ORF72* gene mutation consisting in a hexanucleotide expansion of the sequence GGGGCC (G_4_C_2_) present in the first intron has been associated to ALS and FTD in 2011 (Renton et al. 2011; DeJesus-Hernandez et al. 2011). The physiological size of the expansion can reach 24 repeats, whereas above 30 repeats it is considered pathological. The pathological length of expansion is very heterogenous; indeed, patients generally present hundreds or even thousands of repeats (DeJesus-Hernandez et al. 2011; Gijselinck et al. 2016). Still, the length of the expansion can differ in brain and blood tissues of the same patient. This mosaicism is due to the instability of the repeat number (van Mossevelde et al. 2017). The mutation has an autosomal-dominant transmission and is associated to 40% of fALS, 80% of ALS/FTD, and 25% of familial FTD cases (Gijselinck et al. 2016; van Mossevelde et al. 2017). The mutation leads to both GOF and LOF that together concur in the onset of the pathology. The LOF is due to protein haploinsufficiency that is triggered by the decrease in gene transcription caused by altered RNA structures and by hypermethylation of the promoter (Haeusler et al. 2014; Gijselinck et al. 2016; Esanov et al. 2017). The GOF is associated to the toxicity of the RNA repeats and the formation of abnormal dipeptide-repeat proteins (DRPs). The RNA repeats are transcribed bidirectionally forming sense and antisense transcripts. These transcripts are instable and form nuclear RNA foci that sequester RNA-binding proteins preventing their functionality (Gendron et al. 2013; Barker et al. 2017). In addition, guanosine-rich RNA repeats tend to fold in a stable secondary structure, known as G-quadruplex, that deleteriously interacts with splicing factors causing splicing errors (Reddy et al. 2013; Conlon et al. 2016). The translation of DPR sense and antisense transcripts occurs in an unconventional ATG-independent mechanism and results in the formation of five DRPs constituted of glycine-alanine (GA), glycine-proline (GP), glycine-arginine (GR), proline-arginine (PR), and proline-alanine (PA) (Zu et al. 2013). All DPRs are found in the brain tissue and spinal cord of patients and exert their toxicity by sequestering proteins, impairing ribosome biogenesis, and altering translation. Specifically, the DPRs poly-PR and poly-GR alter nuclear transport (Freibaum and Taylor 2017; Hayes et al. 2020). In addition, poly-GR also interferes with SGs formation and degradation (Chew et al. 2019). Both GOF and LOF concur in alteration of cellular proteostasis. A sign of altered proteostasis stands in TDP-43 mislocalization and aggregation, which is a hallmark of ALS and FTD C9ORF72 patients (Cook et al. 2020). Another sign of altered proteostasis in C9ORF72-patients is the presence of SQSTM1/p62 and ubiquitin-positive inclusions that frequently also contain DPRs (Mann et al. 2013).

C9ORF72 animal models were developed to mimic LOF and GOF. For what concerns C9ORF72 role in autophagy, transient reduction of *C9orf72* expression in conditional knock-out mice in neurons does not cause alterations in behavior or motor phenotype (Koppers et al. 2015), whereas constitutive *C9orf72* knock-out animals present dysregulation of the immune system probably associated to autophagy dysfunction, reduced survival, and mild motor and cognitive phenotypes (Jiang et al. 2016; Atanasio et al. 2016). Overall, data on animals support the theory that loss of C9ORF72 function alone is not sufficient to develop ALS. On the other hand, models of GOF present neuronal alterations.

### CHMP2B

Charged multivesicular body protein 2B (CHMP2B) is encoded by the gene *CHMP2B* (chromosome 3p11.2). It is an evolutionarily conserved protein playing a key role in the assembly of the endosomal sorting complex required for transport III (ESCRT-III; Fig. 4). The ESCRT-III machinery is implicated in the generation of multivesicular bodies (MVBs), endosomal sorting, and autophagy in several tissues, including all brain regions (Skibinski et al. 2005; Rusten and Stenmark 2009; Hurley and Hanson 2010; Henne et al. 2013).

When inactive, CHMP2B is found in an autoinhibited conformation masking the N-terminal sites required to recruit the ESCRT-III complex. Activation occurs through the interaction of CHMP2B C-terminal domain with the ATPase vacuolar protein sorting-associated protein 4 (VPS4) and triggers the assembly of the ESCRT-III and VPS4 complexes on the endosomal membrane. VPS4 ATPase activity is then required to let intraluminal vesicles bud from endosomal membranes, hence generating MVBs, and to finally dissociate ESCRT-III proteins from one another for MVB generation (Fig. 4). (Schmidt and Teis, 2012).

Apart from being involved in MVB maturation, CHMP2B also participates to lysosomal membrane repair and to autophagy together with the other components of the ESCRT-III machinery (Krasniak and Ahmad 2016; Radulovic et al. 2018). Loss of ESCRT genes was demonstrated to correlate with autophagosome accumulation in yeast (Roudier et al. 2005), *Drosophila* (Rusten et al. 2007), and mammals (Komatsu et al. 2006), implying a key role played by ESCRT proteins in autophagosome-lysosome fusion. Even though the mechanism through which CHMP2B is involved in autophagy is still not clearly defined, *CHMP2B* mutations are associated to important signs of autophagy impairment.

The first *CHMP2B* mutation linked to neurodegeneration was found in 1995 in a Danish family affected by autosomal-dominant FTD and was characterized as a substitution occurring at exon 6 splice acceptor site (Brown et al. 1995; Gydesen et al. 2002; Skibinski et al. 2005). Such mutation determines the production of two mutant CHMP2B forms, one including intron 5 in the protein sequence and producing a truncated CHMP2B isoform with a valine residue replacing the last 36 amino acids of the wild-type protein (CHMP2B^Intron5^) and the other generating a 29-amino acid nonsense sequence (CHMP2B^Δ10^) (Skibinski et al. 2005). Additional *CHMP2B* mutations were later identified also in fALS and ALS/FTD (Parkinson et al. 2006; Cox et al. 2010; van Blitterswijk et al. 2012c; Narain et al. 2018).

Truncated CHMP2B mutants are incapable of autoinhibition and tend to associate to the rest of the ESCRT-III machinery at a higher frequency compared to wild-type CHMP2B. This abnormal interaction leads to the formation of aberrant complexes that remain associated to endosomal membranes, thus preventing autophagosome-lysosome fusion (Lee et al. 2007; Han et al. 2012). Indeed, CHMP2B^Intron5^ was shown to induce the accumulation of ubiquitin-positive puncta and/or SQSTM1/p62- and LC3-positive vacuoles in cells (Lee et al. 2007; Filimonenko et al. 2007; West et al. 2020). Autophagosome accumulation was also observed in murine and *Drosophila* CHMP2B^Intron5^ models (Ghazi-Noori et al. 2012; Vernay et al. 2016; West et al. 2020) and even in patient-derived fibroblasts and cortical tissue (Urwin et al. 2010), supporting the involvement of autophagic flux blockage in CHMP2B^Intron5^-related pathogenesis. Additionally, CHMP2B^Intron5^ was evidenced to localize on accumulating endosomes positive for the small GTPases RAB4, RAB5, and RAB7 in both *Drosophila* and rat primary neurons (West et al. 2020). Similar observations were made in postmortem brains of fALS patients bearing *CHMP2B* mutations (T104N, I29V, Q206H), which displayed an accumulation of autophagosomes positive for SQSTM1/p62 and LC3-II (Parkinson et al. 2006; Cox et al. 2010), and the T140N CHMP2B mutant was also shown to localize with other ESCRT-III subunits in RAB5- and RAB7-positive endosomes in cortical neurons (Han et al. 2012). These data support the involvement of endolysosomal and autophagosomal activity dysfunction in CHMP2B-related neurodegeneration.

### DCTN1

Dynactin subunit 1 (DCTN1, or p150^Glued^) is the largest subunit of the dynactin complex and is encoded by the gene *DCTN1* (chromosome 2p13.1). The dynactin machinery is composed of at least 11 proteins organized into more than 20 subunits (Urnavicius et al. 2015). The dynactin complex interacts with dynein to increase its processivity in microtubule-based retrograde transport and acts as a multifunctional adaptor between dynein and specific cargos, including vesicles, organelles, cytosolic proteins, and mRNA (Karki and Holzbaur 1999; Culver-Hanlon et al. 2006). Inside the dynactin complex, DCTN1 forms dimers that interact with dynein intermediate chains and bind to microtubules through its N-terminal cytoskeletal-associated protein/glycine-rich (CAP-Gly) domain (Vaughan and Vallee 1995; Karki and Holzbaur 1995; Culver-Hanlon et al. 2006).

Considering autophagy, DCTN1 plays a central role in dynein-mediated retrograde transport of autophagosomes and lysosomes to the perinuclear region (Fig. 3). DCTN1 was indeed shown to interact with Hermansky-Pudlak syndrome 6 (HSP6), a protein that is strictly required to drive lysosomes to the microtubule organizing center for degradation (Li et al. 2014).

Based on the current knowledge, it is still unclear whether *DCTN1* mutations promote ALS development through GOF or LOF mechanisms. The first pathogenetic mutation ever identified in *DCTN1* sequence was an autosomal dominant missense associated to a form of hereditary motor neuropathy that primarily affectes lower MNs (HMN7B) (Puls et al. 2003). Such mutation produces a G59S substitution in DCTN1 CAP-Gly domain that alters the folding of DCTN1 microtubule-binding domain, reducing the affinity of the mutant protein for microtubules. G59S DCTN1 tends to misfold and form neurotoxic aggregates that were shown to sequester dynein, mitochondria, and TDP-43 (Puls et al. 2003, 2005; Levy et al. 2006; Deshimaru et al. 2021). Additional *DCTN1* mutations lying both in DCTN1 CAP-Gly domain and in its dynein-binding domain have then been identified in ALS (Münch et al. 2004; Stockmann et al. 2013; Liu et al. 2014, 2017), including those forms with an FTD component (Münch et al. 2005). Several in vitro studies were performed to characterize the impact of ALS-related DCTN1 mutations on the protein. While some DCTN1 mutants did not evidence any alterations in protein conformation or distribution (Münch et al. 2004; Dixit et al. 2008; Stockmann et al. 2013), other variants identified in sALS patients were shown to form aggregates or abnormal filamentous structures upon overexpression in primary rat MNs (Stockmann et al. 2013). However, most of the *DCTN1* mutations causing an aberrant protein behavior are characterized by uncertain patterns of inheritance and can be found also in control populations, suggesting that they might represent risk factors predisposing to ALS, rather than overt pathogenetic variants (Stockmann et al. 2013).

Analyses performed by Jiang and colleagues on postmortem samples revealed that both upper and lower MNs of sALS patients display lower *DCTN1* mRNA levels compared to non-ALS controls starting from very early stages of disease progression (Jiang et al. 2005, 2007). Similarly, Kuźma-Kozakiewicz and colleagues evidenced increased *DCTN1* mRNA levels but reduced DCTN1 protein levels in the motor cortex of sALS patients compared to the sensory cortex, suggesting that in sALS MNs DCTN1 depletion occurs and cannot be restored by gene expression upregulation, probably due to inefficient translation (Kuźma-Kozakiewicz et al. 2013). In contrast, while homozygous *Dctn1* knock-out or G59S DCTN1 knock-in was proven to be embryonic lethal in mice, animals deprived of one *Dctn1* allele did not develop MN degeneration (Lai et al. 2007), and neuron-specific *Dctn1* knock-out mice only began manifesting neurodegeneration symptoms at 18 months of age (Yu et al. 2018). On the other hand, two heterozygous G59S DCTN1 knock-in strains generated by distinct research groups displayed halved DCTN1 protein levels and no sign of mutant protein aggregation in brain and spinal cord, suggesting that the G59S substitution might lead to rapid protein degradation in vivo, but at the same time, mice started manifesting muscle atrophy due to spinal MN loss at 10 months of age. These symptoms were accompanied by astrogliosis and by the accumulation of cytoskeletal and synaptic vesicle proteins at neuromuscular junctions, probably as a consequence of disrupted retrograde transport (Levy et al. 2006; Lai et al. 2007). Moreover, a third heterozygous G59S DCTN1 murine strain evidenced fast lower MN loss paralleled by impaired ER-to-Golgi vesicular trafficking and by the accumulation of LC3-II- and ubiquitin-positive DCTN1 aggregates in MN cell bodies hinting at an involvement of dysfunctional retrograde transport of autophagosomes in neuronal death (Laird et al. 2008). In line with these observations, knockout of the *DCTN1* ortholog in *C. elegans*, *dnc-1*, was associated to immature autophagosome accumulation in adult MNs, fostering their degeneration and leading to severe motor deficits that recapitulate ALS symptomatology (Ikenaka et al. 2013). These conflicting data suggest that further studies are required to gain better insight into the pathogenetic mechanisms associated to *DCTN1* mutations in neurodegeneration.

### FIG4

Factor-induced gene 4 (FIG4) is a magnesium-dependent phosphatase encoded by the gene *FIG4* (chromosome 6q21). *FIG4* mediates phosphatidylinositol-3,5-bisphosphate (PI(3,5)P2) conversion to phosphatidylinositol-3-phosphate (PI(3)P) on the cytosolic side of endosomal membranes, where *FIG4* localizes through the interaction with the scaffolding protein VAC14 (Rudge et al. 2004; Sbrissa et al. 2007; Jin et al. 2008). PI(3)P and its derivatives are central players in endosomal transport and autophagy, with different PI3-phosphate pools generated by distinct kinases and phosphatases localized to different endosomal compartments (Di Paolo and De Camilli 2006). Specifically, PI(3,5)P2 is produced by the kinase 1-phosphatidylinositol 3-phosphate 5-kinase (PIKfyve), which joins the complex formed by FIG4 and VAC14 on endosomal membranes, and this modified phospholipid acts as a key signal for the retrograde trafficking of endosomes (Sbrissa et al. 2007; Ferguson et al. 2009). Conversion of PI(3)P to PI(3,5)P2 is also essential for endosomal maturation since PI(3,5)P2 modulates cargo degradation in late endosomes/lysosomes (Odorizzi et al. 1998; Rutherford et al. 2006; Zhang et al. 2007). Therefore, the PIKfyve-FIG4-VAC14 ternary complex plays a central role in regulating endosomal metabolism (Fig. 4).

The first mutation identified in *FIG4* is an insertion of the 5.5-kb retrotransposon *ETn2β* into intron 18 of *Fig4* murine gene, which caused alterations in *Fig4* mRNA processing, therefore resulting in a substantial reduction in Fig4 protein levels. Animals affected by this autosomal recessive mutation displayed resting tremor accompanied by diluted pigmentation and were thus referred to as “pale tremor” mice. This phenotype is consistent with Charcot-Marie-Tooth (CMT) axonal neuropathies; notably, recessively inherited *FIG4* mutations were subsequently identified in CMT4J patients (Chow et al. 2007). ALS-associated *FIG4* mutations include truncating mutations, missenses, and mutations in splice sites and are all predicted to cause loss of protein function, similarly to that observed in pale tremor mice and CMT4J patients (Chow et al. 2007, 2009; Osmanovic et al. 2017). While *FIG4* mutations are reported to be responsible for 1–3% of ALS cases among European patients, no deleterious *FIG4* variants have been found in larger ALS groups, and some of the variants isolated in the European cohort display reduced penetrance (Osmanovic et al. 2017), so that further investigation is required.

Animal models have been exploited to better characterize the role of *FIG4* mutations in endolysosomal trafficking. Concerning pale tremor mice, the relevant reduction in Fig4 protein levels dependent on *ETn2β* retrotransposition determined a strong decrease in PI(3,5)P2 levels accompanied by the accumulation of large vacuoles harboring ubiquitinated proteins and positive for SQSTM1/p62, LC3-II, and the late-stage endosomal markers lysosomal-associated membrane proteins 1 and 2 (LAMP-1 and LAMP-2) in murine neurons and astrocytes (Ferguson et al. 2009; Lenk et al. 2011). Comparable accumulation of enlarged lysosomes causing the rapid development of severe brain degeneration was observed in both constitutive and neuron-specific *Fig4* knock-out murine models (Ferguson et al. 2012), while in *Drosophila Fig4* loss correlated with motility impairments and shorter lifespan (Bharadwaj et al. 2016; Kyotani et al. 2016). The decrease in PI(3,5)P2 levels associated to *Fig4* loss is hypothesized to depend on reduced PIKfyve activity, since FIG4 is required to stabilize the interaction between PIKfyve and VAC14 (Botelho et al. 2008).

### KIF5A

Kinesin 5A (KIF5A) is a neuron-specific kinesin heavy chain encoded by the gene *KIF5A* (chromosome 12q13.3) (Aizawa et al. 1992). Kinesins are ATP-dependent molecular motors that transport cargo along microtubule tracks in the anterograde direction, from the center of the cell to its periphery (Vale et al. 1985; Brady 1985). KIF5A comprises an N-terminal motor domain involved in microtubule binding and ATP hydrolysis, a stalk domain required for homodimerization and interaction with kinesin light chains, and a C-terminal tail domain which mediates interaction with cargos and adaptor proteins (Miki et al. 2005; Hirokawa and Noda 2008). Among KIF5A cargos, lysosomes can be found, conferring KIF5A a role in the autophagic pathway (Fig. 3) (Liu et al. 2021).

*KIF5A* was found mutated in ALS in 2018, with genome-wide analyses identifying both low- and high-risk mutations (Brenner et al. 2018; Nicolas et al. 2018). Mutations in *KIF5A* had been previously linked to other neurodegenerative or neurodevelopmental disorders. Interestingly, mutations targeting different KIF5A domains give rise to distinct phenotypes, with minimal overlapping. Specifically, mutations targeting KIF5A motor or stalk domains cause hereditary spastic paraplegia and CMT2 (Reid et al. 2002; Fichera et al. 2004; Crimella et al. 2012), while mutations falling in its C-terminal tail are associated to neonatal intractable myoclonus as well as ALS (Rydzanicz et al. 2017; Brenner et al. 2018; Nicolas et al. 2018).

Recently, de novo frameshift mutations causing exon 27 skipping (ΔExon27) and downstream elongation of the KIF5A tail have been reported to abolish KIF5A ability to perform autoinhibition, leading to aberrant mitochondrial transport along axons, and to enhance mutant KIF5A interaction with SQSTM1/p62 (Baron et al. 2022). Therefore, neurodegeneration caused by ΔExon27 KIF5A mutations seems to be linked to a toxic GOF disrupting neuronal trafficking and homeostasis. Since lysosomes are part of KIF5A cargos (Liu et al. 2021), the axonal transport alterations implicated in KIF5A-related ALS pathogenesis might tamper with the autophagic flux, too. Further studies are required to better elucidate the contribution of *KIF5A* mutations to ALS.

### OPTN

OPTN is a multifunctional adaptor protein encoded by the *OPTN* gene (chromosome 10p13). It is highly expressed in brain and skeletal muscle (Rezaie et al. 2002; De Marco et al. 2006) and takes part in a wide variety of cellular processes, such as NF-κB activation, viral sensing, Golgi maintenance, and autophagy (Markovinovic et al. 2017). OPTN acts as an autophagy receptor by binding substrates, including damaged mitochondria, through its C-terminal ubiquitin-binding region of ABIN proteins and NEMO (UBAN) domain and delivers them to phagophores by interacting with LC3, thanks to its LIR domain (Figs. 1 and 2) (Wild et al. 2011; Wong and Holzbaur 2014). Subsequently, it links to myosin VI to promote autophagosome fusion with lysosomes (Tumbarello et al. 2012).

OPTN role as an autophagy receptor makes it a neuroprotective factor. Indeed, OPTN is sequestered into aggregates formed by mutant proteins in several neurodegenerative disorders (Maruyama et al. 2010; Hortobágyi et al. 2011; Osawa et al. 2011), and its deletion promotes aggregates accumulation (Korac et al. 2013). Additionally, OPTN interacts with SOD1 aggregates through its C-terminal coiled-coil domain, therefore in an ubiquitin-independent fashion (Korac et al. 2013).

*OPTN* mutations were initially associated to primary open-angle glaucoma (Rezaie et al. 2002; Minegishi et al. 2016) and were found to impair the autophagic flux. For example, retinal cells harboring OPTN E50K were shown to be characterized by defects in phagophore formation and by autophagy inhibition upon amino acid starvation (Chalasani et al. 2014). For what concerns ALS, more than 40 *OPTN* variants have been reported both in sALS and in fALS, with a limited number of mutations being shared between populations of different ethnicity (Maruyama et al. 2010; Del Bo et al. 2011; Tümer et al. 2012; van Blitterswijk et al. 2012b; Iida et al. 2012; Beeldman et al. 2015; Gotkine et al. 2021). ALS-related *OPTN* mutations are mainly missenses, truncations, and exon 5 deletions. A LOF mechanism is hypothesized for truncating mutations since mRNA decay and loss of OPTN immunoreactivity were reported in patient spinal cord (Maruyama et al. 2010; Iida et al. 2012; Gotkine et al. 2021). Regarding missense variants, they tend to cluster around OPTN UBAN domain (Maruyama et al. 2010). The best characterized one is E478G OPTN, which is defective in ubiquitin binding (Wild et al. 2011) and was indeed reported to lose the ability to associate with polyubiquitinated inclusions and mitochondria to drive them to autophagy (Korac et al. 2013; Wong and Holzbaur 2014). The E478G mutant also sequesters wild-type OPTN into oligomers upon overexpression, limiting the OPTN pool available to guide phagophore formation (Shen et al. 2015) and forming inclusion bodies in sALS neurons (Maruyama et al. 2010).

Furthermore, E478G and G398X OPTN were shown to be unable to bind to myosin VI, determining the accumulation of immature autophagosomes triggering ER and Golgi stress (Sundaramoorthy et al. 2015).

Animal models recapitulate most observations on ALS-associated *OPTN* mutations made in vitro. OPTN ortholog knock-down in zebrafish is associated to motor axonopathy symptoms resembling models of SOD1 pathology (Korac et al. 2013). Moreover, 470T Optn mice mimic human OPTN truncations and reduced protein levels following mRNA decay are reported for such animal model (Munitic et al. 2013). Finally, the murine equivalent of the human E478G mutation (D477N Optn) is similarly unable to bind ubiquitin (Gleason et al. 2011). Despite the wide variety of data collected about *OPTN* mutations in ALS, the pathogenetic mechanisms causing autophagy impairments lying behind them are still to be fully characterized.

### SQSTM1/p62

SQSTM1/p62 is a multifunctional adaptor protein encoded by the gene *SQSTM1* (chromosome 5q35.3). It is highly expressed in spinal cord MNs (Keller et al. 2012) and is implicated in several cellular processes, including NF-κB activation, apoptosis, and proteostasis maintenance (Rea et al. 2013). Concerning the latter process, SQSTM1/p62 acts as an autophagy receptor similarly to OPTN, therefore by sequestering ubiquitinated proteins via its C-terminal ubiquitin-binding domain (UBD), named ubiquitin-associated (UBA) domain, and contemporarily interacting with LC3 through its LIR to deliver substrates to autophagosomes (Figs. 1 and 2) (Bjørkøy et al. 2005; Pankiv et al. 2007; Ichimura et al. 2008). The N-terminal Phox and Bem1p-1 (PB1) ubiquitin-like domain allows SQSTM1/p62 to oligomerize, and both the PB1 and the UBA domains are involved in the interaction with other autophagy receptors, like OPTN and neighbor of BRCA1 gene 1 (NBR1), required to better coordinate autophagosome formation (Johansen and Lamark 2011). SQSTM1/p62 is also able to drive ubiquitinated substrates to proteasomal degradation, thanks to its ubiquitin-like domains (Seibenhener et al. 2004; Babu et al. 2005; Geetha et al. 2008). Evidence supports the hypothesis that SQSTM1/p62 might be a neuroprotective factor, once again similarly to OPTN. Firstly, it is upregulated at the onset of neuronal apoptosis, when ubiquitinated proteins accumulate in response to cell damage (Kuusisto et al. 2001). Moreover, it is found within ubiquitin-positive inclusions formed by mutant proteins in several neurodegenerative disorders (Zatloukal et al. 2002; Arai et al. 2003; Mizuno et al. 2006; King et al. 2013), underlining the central role this autophagy receptor plays in PQC. SQSTM1/p62 is also responsible for driving mutant SOD1 to autophagosomes through an ubiquitin-independent interaction mediated by its SOD1 mutant interaction region (SMIR) (Gal et al. 2009). Lastly, mice deprived of *Sqstm1* display tau hyperphosphorylation and memory impairments (Babu et al. 2008), both hallmarks of Alzheimer’s disease, while *Sqstm1* knock-down in zebrafish determines the development of locomotory impairments associated with autophagy defects and MN axon shortening (Lattante et al. 2015).

*SQSTM1* mutations were firstly identified in 2002 in patients suffering from Paget disease of bone (PDB), a chronic progressive skeletal disorder. PDB-causing *SQSTM1* mutations mainly target SQSTM1/p62 UBA domain, with the P392L substitution being the most frequent among them (Laurin et al. 2002). Mutations spanning the whole *SQSTM1* sequence, including its promoter, have thereafter been related to fALS, sALS, and ALS/FTD (Rubino et al. 2012; Teyssou et al. 2013; Hirano et al. 2013; Shimizu et al. 2013; Le Ber et al. 2013; Chen et al. 2014; Kwok et al. 2014), and in most cases they have been associated with TDP-43 pathology (van der Zee et al. 2014). Since many ALS-related *SQSTM1* variants were also found at relatively high frequencies in control populations, it is still unclear whether they are actually causative of the phenotype, as it remains to be determined how mutations in the same gene give rise to such distinct phenotypes as ALS and PDB (Rea et al. 2014). Nonetheless, the body of evidence connecting ALS-associated *SQSTM1* mutations to specific impairments of SQSTM1/p62 functions in autophagy is growing. For example, the L341V substitution in SQSTM1/p62 LIR sequence was shown to cause a strong decrease in its interaction with LC3-II, thus tampering with the ability of the autophagy receptor to deliver cargo to forming phagophores (Goode et al. 2016). Additionally, some studies have confirmed the presence of SQSTM1/p62-positive inclusions along with increased levels of the protein in spinal MNs of *SQSTM*1 mutation carriers and found frontal cortical atrophy associated with these aggregates, thus providing further evidence of pathologic overlap between ALS and FTLD (Teyssou et al. 2013; Le Ber et al. 2013).

Until now, mechanistic studies on *SQSTM1* mutations have mainly concentrated on the P392L substitution. Overexpression of P392L SQSTM1/p62 was shown to enhance autophagosome formation in the osteoclasts of mice harboring the murine equivalent of the P392L mutation (Daroszewska et al. 2011). Additionally, rescue of the locomotor defects displayed by zebrafish upon *Sqstm1* downregulation could be achieved through overexpression of human wild-type SQSTM1/p62, but not of the P392L mutant. Amelioration of the phenotype was also evidenced upon administration of the autophagy inductor rapamycin, which further supports the hypothesis of a LOF being connected to the P392L substitution (Lattante et al. 2015). Interestingly, the P392L mutation was later found in ALS patients (Teyssou et al. 2013; Le Ber et al. 2013; Kwok et al. 2014), but its effect on MNs has not been characterized yet.

Finally, *Sqstm1* depletion was demonstrated to exacerbate H46R SOD1 mice ALS phenotype, with shorter lifespan and accelerated MN degeneration and weight loss (Hadano et al. 2016).

### TBK1

TANK-binding kinase 1 (TBK1) is a serine/threonine kinase encoded by the gene *TBK1* (chromosome 12q14.2). TBK1 is highly expressed in neuronal cells belonging to the cerebral cortex, the hippocampus, and the lateral ventricle (Uhlén et al. 2015).

TBK1 was first isolated as OPTN binding partner through yeast two-hybrid screening (Morton et al. 2008). In fact, it phosphorylates OPTN in its UBAN domain, promoting its binding to LC3-II and to ubiquitinated substrates to enhance the autophagic flux (Oakes et al. 2017). OPTN phosphorylation also enforces its role in TBK1 activation, which generates a positive feedback loop between TBK1 and OPTN activation (Lazarou et al. 2015; Heo et al. 2015). TBK1 exerts a similar modulatory activity on nuclear domain 10 protein 52 (NDP52) and SQSTM1/p62, two other autophagy adaptors, increasing their affinity for the ubiquitin chains of their UBDs through phosphorylation (Thurston et al. 2009; Pilli et al. 2012; Li et al. 2018a). TBK1-mediated OPTN, NDP52, and SQSTM1/p62 phosphorylation also promotes their association with damaged mitochondria to support mitophagy (Lazarou et al. 2015; Heo et al. 2015; Richter et al. 2016; Moore and Holzbaur 2016). Additionally, this kinase targets the C9ORF72-coupled GEF SMCR8, leading to C9ORF72 activation and therefore promoting autophagy initiation (Fig. 1) (Sellier et al. 2016). Moreover, TBK1 plays a role in autophagosome maturation, probably by interacting with the small GTPase RAB8b. TBK1 silencing was indeed shown not to interfere with autophagosome formation but to prevent autophagosome-lysosome fusion (Wang et al. 2018). Finally, microtubule-binding proteins required to trigger retrograde transport of autophagosomes and autolysosome formation are among TBK1 substrates, too (Oakes et al. 2017).

*TBK1* mutations were firstly identified in ALS in 2015 through whole-exome sequencing of large patient cohorts of European ethnicity. Subsequently, additional studies confirmed the role of *TBK1* in ALS pathogenesis, including ALS/FTD (Le Ber et al. 2015; Williams et al. 2015; Shu et al. 2016; Tsai et al. 2016; Borghero et al. 2016; van Rheenen et al. 2016). To date, more than 90 *TBK1* mutations have been reported (Abramzon et al. 2020), and while they are rare in sALS patients, they account for around 3–4% of fALS and ALS/FTD cases (Cirulli et al. 2015; Freischmidt et al. 2015; Gijselinck et al. 2015). Most identified *TBK1* variants are missense mutations with uncharacterized pathogenic effects (Oakes et al. 2017). On the other hand, nonsense and frameshift *TBK1* mutations associated to the deletion of its second coiled-coil domain (CCD2) are reported to alter TBK1 ability to interact with adaptor proteins that regulate its localization and activation of downstream signaling pathways (Ryzhakov and Randow 2007). Nonsense and frameshift *TBK1* mutations also correlate with reduced TBK1 expression as a consequence of mRNA decay (Freischmidt et al. 2015; Brenner et al. 2019). Thus, the pathogenetic mechanisms underlying TBK1-related ALS cases are considered to depend on the loss of TBK1 function.

Abolishment of the interaction between TBK1 and OPTN by *TBK1* mutations seems to play a prominent role in ALS pathogenesis. Indeed, an ALS-related TBK1 mutant deprived of amino acids 690–713 in the CCD2 domain was shown to be unable to bind OPTN when transiently overexpressed in HEK293T cells while preserving interaction with other TBK1 protein partners (Freischmidt et al. 2015). The same was demonstrated for additional TBK1 mutants (Richter et al. 2016; Moore and Holzbaur 2016; Li et al. 2018a; de Majo et al. 2018). Moreover, E696K TBK1 was shown to correlate with decreased recruitment of OTPN and LC3 to damaged mitochondria, leading to mitochondrial dysfunction and accumulation (Moore and Holzbaur 2016). Finally, *TBK1* mutations were shown to correlate with the formation of inclusions positive for TDP-43, ubiquitin, and SQSTM1/p62 in MNs and glial cells of ALS and FTD patients (Van Mossevelde et al. 2016), further reflecting autophagy disruption. Taken together, these data support the hypothesis that the concerted activity of TBK1 and OPTN in autophagy and mitophagy is essential to prevent neurodegeneration.

Animal models have been generated to better investigate TBK1 role in ALS. While heterozygous *Tbk1* knock-out mice did not develop MN degeneration or autophagy impairments (Brenner et al. 2019), loss of murine *Tbk1* resulted embryonic lethal (Bonnard et al. 2000), and animals undergoing neuron-specific *Tbk1* deletion displayed loss of cortical synapses, dendrite dysmorphism, and accumulation of neurofibrillary tangles correlating with motor and cognitive defects reminiscent of ALS/FTD (Duan et al. 2019). Additionally, loss of one *Tbk1* allele in a G93A SOD1 murine model accelerated autophagy dysfunction and muscle denervation during the early stage of ALS, but at the same time, it extended mouse survival by reducing neuroinflammation at later stages (Brenner et al. 2018). A comparable disease course was observed in G93A SOD1 mice harboring *Tbk1* mutations that impair its kinase activity (Gerbino et al. 2020). Therefore, these findings seem to indicate that loss of *TBK1* function does not only lead to ALS development but can also modify the course of the pathology.

### TUBA4A

α-tubulin isoform 4a (TUBA4A) is encoded by the gene *TUBA4A* (chromosome 2q35). It is ubiquitously expressed but particularly enriched in neurons (Rustici et al. 2013; Smith et al. 2014). α-and β-tubulins heterodimerize to form microtubules, cytoskeletal scaffolds along which kinesin- and dynein-mediated transport occurs in cells (Fig. 3).

*TUBA4A* mutations were firstly identified in familial ALS and ALS/FTD in 2014 based on exome sequencing performed on a large cohort of European and American patients (Smith et al. 2014). In the following years, additional *TUBA4A* mutations were found in other European and Asian cohorts (Dols-Icardo et al. 2016; Perrone et al. 2017; Li et al. 2018b) and even among sporadic ALS and ALS/FTD patients (Pensato et al. 2015). Differently from other tubulin genes that are highly expressed during brain development and that are therefore found mutated in neurodevelopmental disorders, *TUBA4A* expression increases with aging (Tischfield et al. 2011; Hersheson et al. 2013), which might explain why its mutations are associated to late-onset neurodegeneration (Smith et al. 2014; Clark et al. 2016) and may account for TUBA4A contributions to ALS progression. The frequency of *TUBA4A* mutations is anyway low in all tested populations so far, making them a rare cause of ALS (Chia et al. 2018).

Most *TUBA4A* mutations found in ALS cluster in the protein domain involved in the interaction with other tubulins and with the molecular motors kinesin and dynein (Howes et al. 2014) and are therefore predicted to tamper with microtubule stability and microtubule-based transport in cells. To date, the best characterized ALS-associated TUBA4A mutant is the truncated W407X variant, which displays impaired α/β-tubulin dimer formation and incorporation into microtubules accompanied by aggregation propensity when expressed in primary MNs (Smith et al. 2014).

Concerning autophagy, disruption of microtubule dynamics related to *TUBA4A* mutations might negatively reflect on autolysosome formation, which is dependent on dynein-mediated retrograde transport (Rademakers and van Blitterswijk 2014). Mutant TUBA4A aggregation might further worsen autophagy impairment; however, up to now, no data are available to confirm this hypothesis except for the fALS-associated W407X mutant (Smith et al. 2014). The generation of animal models could help in characterizing the pathogenetic mechanisms associated to *TUBA4A* mutations in ALS.

### UBQLN2

Ubiquilin-2 (UBQLN2) is one of the four members of the ubiquilin (UBQLN) family in the human genome encoded by the *UBQLN2* gene located on chromosome Xp11.21 (Kaye and Shows 2000). It is expressed in various tissues, but the highest expression levels are found in muscles and brain (Lin et al. 2021). UBQLN2 localizes mainly in the cytoplasm where it functions in the maintenance of proteostasis. UBQLN2 presents an ubiquitin-like (UBL) domain at the N-terminus, which interacts with the regulatory cap of the proteasome, and a UBA domain at the C-terminus, which recognizes polyubiquitin chains present on substrates; the UBQLN2 central domain is characterized by the presence of four stress-induced protein 1 (STI-1)-like motifs, which bind to heat shock proteins through a still unclear mechanism, and a proline-rich repeat domain containing 12 PXX repeats whose function is unknown (Kaye et al. 2000; Walters et al. 2002; Ko et al. 2004; Deng et al. 2011). 

UBQLN2 activity mainly consists in promoting the disposal of misfolded or redundant proteins through the UPS or autophagy. The UBL and UBA domains permit the shuttling of ubiquitinated proteins to the UPS. UBQLN2 substrates are cytosolic and ER proteins. Indeed, through its binding with ER membrane proteins as ubiquitin regulatory X domain-containing protein 8 protein (Ubxd8) and homocysteine-induced ER protein (Herp), UBQLN2 specifically promotes the disposal of altered ER proteins. UBQLN2 interaction with autophagy is more complex. In the first place, UBQLN2 is implicated in autophagy initiation; indeed, it regulates mTORC1 activity. Loss of UBQLN2 activity prevents mTORC1-mediated autophagy inhibition resulting in an increased autophagy induction (Şentürk et al. 2019). Furthermore, UBQLN2 interacts indirectly with LC3 through its UBA domain promoting autophagosome formation (N’Diaye et al. 2009), and it was found also to interact with OPTN, confirming a role of UBQLN2 in the first steps of the autophagic pathway (Figs. 1 and 2) (Osaka et al. 2015). Recent data showed a role of UBQLN2 also in lysosomal activity by regulating its acidification. Indeed, different groups presented two parallel mechanisms through which UBQLN2 interacts with subunits of v-ATPase, promoting its formation (Fig. 3) (Şentürk et al. 2019; Wu et al. 2020). Thus, silencing or mutations of *UBQLN2* gene are associated to an increase in autophagy induction that is paralleled with a blockage of autophagosome disposal due to a loss of lysosome activity (Şentürk et al. 2019). 

*UBQLN2* mutations were associated in 2011 to a dominantly inherited, X-linked form of ALS and ALS/FTD (Deng et al. 2011). To date, more than 20 mutations have been detected mainly in the PXX domain, while fewer are present in STI-1-like motifs or between domains [as reviewed in (Renaud et al. 2019)]. *UBQLN2* mutations impair cellular homeostasis with different mechanisms: by triggering proteasome impairment, preventing the delivery to the proteasome of polyubiquitinated substrates, which accumulate leading to further cellular alterations (Chang and Monteiro 2015); by altering different steps of autophagy as previously described; by interacting with TDP-43 and its C-terminal fragments, triggering their aggregation and formation of cytoplasmatic inclusions (Cassel and Reitz 2013; Picher-Martel et al. 2015); and by promoting neuroinflammation through direct interaction with NF-κB, an inflammation transcriptional factor regulator, or through a TDP-43-dependent process (Swarup et al. 2011). Different pathological mechanisms associated with *UBQLN2* mutations result in colocalization of UBQLN2 and TDP-43 cytoplasmic inclusions in the brain and the spinal cord of patients. Of interest, UBQLN2 colocalization with TDP-43 was also present in the spinal cord of sALS patients, underling a central role for UBQLN2 in ALS pathology (Fecto and Siddique 2011).

In vivo models were developed to better understand UBQLN2 pathology. The first model generated was a mouse strain harboring the P497H UBQLN2 substitution under the human UBQLN2 promoter which expressed low protein levels (Gorrie et al. 2014). Because of the limited mutant UBQLN2 expression, this model presented cognitive impairments and hippocampal inclusions but did not show MNs loss. Subsequently, two rat models harboring the same mutation but expressed at higher levels were developed. These models presented impaired autophagy and endocytosis associated with neuronal loss (Wu et al. 2015; Chen et al. 2018). Also, transgenic mice expressing the P520T substitution, which is highly aggressive in humans causing an early ALS/FTD onset (Deng et al. 2011), failed to present neurodegeneration, but only displayed alterations in neuronal proteostasis (Sharkey et al. 2020). Furthermore, mice and rat knock-out models showed late or no neurodegenerative phenotype. Together, these data suggest that UBQLN2 pathology is triggered by both GOF and LOF.

### VAPB

VAMPs-associated protein B (VAPB) is a member of the evolutionarily conserved VAP protein family (Skehel et al. 1995) encoded by the gene *VAPB* (chromosome 20q13.32). VAPs localize on the membrane of the ER and of ER-Golgi intermediates, in close association with vesicle-associated membrane proteins (VAMPs). There, VAPs act as adaptors for the recruitment to the ER surface of cytosolic proteins mainly identified by the two phenylalanines in an acidic tract (FFAT) motif (Loewen et al. 2003). VAP proteins comprise an N-terminal major sperm protein (MSP) domain (Kaiser et al. 2005) involved in target proteins recruitment, a central coiled-coil domain, and a C-terminal hydrophobic sequence anchoring VAPs to the ER membrane. Among VAP interactors, many proteins allowing to put the ER in contact with other organelles can be found. The interaction sites that form through this process are known as membrane contact sites (MCS) and represent regions of communication between organelles achieved in the absence of membrane fusion (Prinz et al. 2020).

Based on these features, VAPB takes part in several cellular processes, among which autophagy can be found. For example, VAPB triggers mitophagy by interacting with the mitochondrial protein tyrosine phosphatase interacting protein 51 (PTPIP51), thus promoting autophagosome formation at the ER-mitochondria interface (Fig. 3) (Gomez-Suaga et al. 2017). Additionally, VABP regulates autophagy through its interaction with RAB3 GTPase-activating protein 1 (RAB3GAP1), which plays a role in autophagy initiation (Spang et al. 2014).

The autosomal dominant P56S substitution was the first and best characterized *VAPB* mutation ever associated to ALS (Nishimura et al. 2004; Funke et al. 2010; Di et al. 2016; Guber et al. 2018). Other *VAPB* mutations were thereafter associated to fALS (Chen et al. 2010; van Blitterswijk et al. 2012a; Sun et al. 2017). The observations made in cellular and animal models of P56S VAPB pathology suggest that both LOF and GOF mechanisms might be implicated in neurodegeneration. Nevertheless, data collected in ALS patient-derived cell models are in contrast with the GOF hypothesis. The P56 VAPB residue is evolutionarily conserved and lies in the MSP domain, in close proximity to the FFAT binding site (Nishimura et al. 2004). The P56S substitution almost entirely prevents VAPB binding to its FFAT-containing cytosolic partners; this contemporarily induces mutant VAPB aggregation both in cellular (Nishimura et al. 2004; Kanekura et al. 2006; Teuling et al. 2007; Suzuki et al. 2009) and in murine ALS models (Tudor et al. 2010; Qiu et al. 2013; Kuijpers et al. 2013a; Aliaga et al. 2013). Aggregating P56S VAPB also sequesters into its inclusions both wild-type VAPB (Kanekura et al. 2006; Teuling et al. 2007; Suzuki et al. 2009) and other proteins involved in membrane trafficking and MCS formation (De Vos et al. 2012; Kuijpers et al. 2013b; Hua et al. 2017). Moreover, increased SQSTM1/p62 and LC3 levels were detected in the spinal cord of P56S VAPB mice, possibly indicating alterations in the autophagic flux (Larroquette et al. 2015). This observation, coupled with the fact that P56S VAPB accumulation was shown to determine profound rearrangement of the affected ER sites in cells (Fasana et al. 2010; Papiani et al. 2012), might provide a link between disrupted VAPB function, ER stress, and autophagy impairments. Indeed, the reduction in ER-mitochondria MCS observed upon VAPB depletion resulted in an enhancement of the autophagic flux in HEK293 cells, probably due to impaired ER-to-mitochondria calcium transfer triggering mitophagy (Gomez-Suaga et al. 2017). At the same time, reduced VAPB protein levels and no signs of VAPB aggregation were detected in sALS spinal cord MNs (Teuling et al. 2007; Anagnostou et al. 2010) and in P56S VAPB fibroblasts, iPSCs, and iPSC-derived MNs (Mitne-Neto et al. 2011). Additionally, despite harboring MN inclusions, most P56S VAPB murine models (Tudor et al. 2010; Qiu et al. 2013; Kuijpers et al. 2013a) did not develop ALS-like motor symptoms, except for the ones overexpressing the highest levels of the transgenic protein (Aliaga et al. 2013). The same occurred for *Drosophila* models harboring the P56S substitution (Sanhueza et al. 2014; Moustaqim-Barrette et al. 2014). At the same time, though, VAPB-null mice develop mild motor dysfunction (Kabashi et al. 2013), suggesting that loss of VAPB function alone might not be sufficient to justify the onset of ALS symptoms in patients. Overall, the contrasting evidence coming from cellular and animal models indicates that further investigation is required to better elucidate the role of *VAPB* mutations in ALS pathogenesis.

### VCP

Valosin-containing protein (VCP) is an ATPase associated with diverse cellular activities (AAA^+^) encoded by *VCP* gene (chromosome 9p13.3). VCP is ubiquitously expressed in tissues (Koller and Brownstein 1987). At cellular level, VCP localizes mainly found in the cytoplasm, while a smaller fraction binds to organelles or localizes in the nucleus (Acharya et al. 1995; Latterich et al. 1995; Madeo et al. 1998; Xu et al. 2011; Ramanathan and Ye 2012). VCP functions assembling in a homo-hexamer. Each monomer presents an N-terminal domain that interacts with adaptors and cofactors; two ATPase domains, D1 and D2, that function respectively in the formation of the homo-hexamer and in accomplishing VCP activity; and a C-terminal domain that cooperates with D2 activity and binds to a small subset of VCP partners (Huyton et al. 2003; Niwa et al. 2012; Chou et al. 2014). 

VCP mechanism of action is to identify and segregate ubiquitinated proteins from different cellular compartment and to enhance their degradation through the UPS or the autophagic pathway. Thanks to the binding of various cofactors and adaptors, VCP has a key role in the maintenance of cellular homeostasis, regulating different cellular pathways as endoplasmic reticulum-associated degradation (Stein et al. 2014), organelle degradation (Tanaka et al. 2010; Papadopoulos et al. 2017), ribosome-associated degradation (Verma et al. 2013), regulation of autophagy (Hill et al. 2021), chaperone activity (Hirabayashi et al. 2001), chromatin-associated degradation (Meerang et al. 2011), NF-κB activation (Dai et al. 1998), and membrane fusion (Amenta et al. 1978). Most of VCP activity is aimed at regulating and preserving cellular proteostasis. To contribute to the maintenance of proteostasis, VCP is involved in the routing of misfolded proteins and aggregates to the UPS or autophagy for their disposal (Figs. 1 and 2). 

VCP role in addressing substrates to autophagy is still not completely clear. However, it was established that VCP interacts with different partners involved in aggresome formation and promotes substrates routing to autophagosomes (Iwata et al. 2005; Boyault et al. 2006). Besides this, VCP regulates different steps of the autophagic pathway [as reviewed in (Ferrari et al. 2022)]. VCP participates in the activation of transcriptional regulators of autophagy, such as NF-κB and TFEB. In particular, VCP promotes the degradation of IκBα that releases NF-κB permitting its activation (Dai et al. 1998). On the other hand, VCP mechanism in the regulation of TFEB is unknown, but when lysosomal damage is induced, the silencing or inhibition of VCP activity stabilizes TFEB activation, suggesting that VCP modulates TFEB action (Arhzaouy et al. 2019). In addition, VCP is involved in autophagy initiation and maturation (Figs. 1 and 2). Recent data showed that VCP promotes the formation of the initiation complex ATG14-VPS34-VPS15-BECN1 through two distinct pathways (Hill et al. 2021). Different studies show that *VCP* mutation impacts on autophagosome maturation; indeed, *VCP* silencing or mutations are correlated with an increase in LC3-II and SQSTM1 protein levels and accumulation of autophagosomes with increased size (Ju et al. 2009; Tresse et al. 2010). Finally, VCP has a key role in regulating lysosome stability and disposal. Studies on muscle tubular lysosomes show that VCP, supported by its cofactor small VCP interacting protein (SVIP), preserves lysosome stability (Fig. 3). Indeed, the inhibition or downregulation of VCP causes lysosomal fragmentation (Johnson et al. 2015). VCP, together with its cofactors phospholipase A2 activating protein (PLAA), UBX domain-containing protein 6 (UBXD1), and ubiquitin thioesterase OTU1 (YOD1), is also essential for damaged lysosome degradation. Indeed, they promote the removal of ubiquitinated proteins exposed on damaged lysosome membrane, an essential step in lysosome disposal (Papadopoulos et al. 2017). 

The essential involvement of VCP in proteostasis is also demonstrated by cellular alterations of VCP-patients affected tissue. *VCP* mutations have been mainly associated in 2004 to inclusion bodies myopathy Paget disease and frontotemporal dementia (IBMPFD), a multisystem proteinopathy, and in 2010 to fALS (Watts et al. 2004; Johnson et al. 2010). To date, more than 40 missense mutations, localized at the interface between N-terminal and D1 domains, were associated to IBMPFD, and almost 20 mutations, localized at N-terminal and D1 interface and in D2, were associated to ALS (Johnson et al. 2010; Mehta et al. 2013; Kenna et al. 2013). VCP-patients present different phenotypes, yet they display similar alterations at cellular level. In skeletal muscles of VCP-patients inclusions positive to ubiquitin and VCP, damaged lysosomes and rimmed vacuoles can be found (Watts et al. 2004; Kimonis and Watts 2005; Ritson et al. 2010). Similarly, in affected neurons cytoplasmatic inclusions positive to VCP and ubiquitin are also found (Kimonis and Watts 2005). Moreover, in both muscles and neurons, TDP-43 mislocalizes and forms inclusions (Ritson et al. 2010).

Animal models recapitulate most observations made on *VCP* mutations in vitro and in VCP-patients. VCP mouse models with *VCP* mutations or conditional knockout (Watts et al. 2004; Johnson et al. 2010) present lower survival with TDP-43 pathology in spinal cord and/or in skeletal muscle, muscle weakness, increase in fibers size that harbor ubiquitinated protein inclusions, and increased autophagy activity (Yin et al. 2012; Nalbandian et al. 2012). VCP mutants-induced neurodegeneration is also visible in *Drosophila* VCP models that overexpress mutations of TER94 (VCP ortholog in *Drosophila*), such as R152H, R188Q, and A229E (corresponding to human VCP mutations R155H, R191Q, and A232E). These models present disruption in nervous system and in muscle tissue that additionally shows specific lysosomal alterations (Chang et al. 2011; Johnson et al. 2015). Together, data obtained from animal models show an impairment in the autophagic pathway, reinforcing VCP essential role in this pathway.

## Conclusions

Research over the past decades has highlighted the central role played by the autophagic pathway in ALS. Autophagy has a determinant role in the disposal of toxic proteins, including the aberrant aggregates formed by mutated proteins in ALS, and damaged organelles, and its failure in keeping under control protein misfolding upon saturating conditions is strictly associated to ALS progression. For this reason, the modulation of the autophagic pathway has been studied by different groups and has been outlined as a target to ameliorate ALS-related pathological conditions. Moreover, as this review summarizes, several ALS-associated mutations are found in genes implicated in all different steps of the autophagic pathway, from transport to protein degradation. Despite the efforts in studying ALS, to date no cure is available, and no therapies that drastically ameliorate life-span have been discovered yet. Thus, by analyzing the physiological role of ALS-related genes involved in autophagy and by evaluating the main experimental data linking mutations in these genes to ALS, it is clear that not all the implications of autophagy dysfunction in ALS have been completely unraveled yet. Indeed, in several cases, the molecular mechanisms explaining the pathogenicity of autophagy-related gene mutations found in ALS are still to be fully deciphered. In some cases, in vitro and cellular analyses might contrast with observations made in patients or in animal models, indicating that further studies are required to fully elucidate the connection between genes involved in the various steps of autophagy and ALS. In addition, this review highlights that alterations in autophagy are directly linked with ALS progression and suggests that a better functionality of autophagy could ameliorate the phenotype. Indeed, various studies on modulation of autophagy have been carried out and are ongoing with promising results. Compounds that positively modulate autophagy, such as trehalose, rapamycin, and colchicine, have been tested in animals or are already in clinical trial (Castillo et al. 2013; Mandrioli et al. 2018, 2019). These compounds stimulate autophagy through different mechanisms that have still to be completely clarified. Potentiation of the autophagic flux in ALS is surely positive in presence of a LOF of autophagy or in presence of impairment of organelles or accumulation of protein aggregates. Contrary, the implication of a potentiation of autophagy in presence of alterations derived by mutations of autophagic components has to be carefully analyzed. In this context, a better understanding of ALS-related genes function and contribution in each step of autophagy is fundamental to fully comprehend the pathological mechanisms and to display novel rescue strategies.
